# Evidence of increased exposure to *Toxoplasma gondii* in individuals with recent onset psychosis but not with established schizophrenia

**DOI:** 10.1371/journal.pntd.0006040

**Published:** 2017-11-06

**Authors:** Robert Yolken, E. Fuller Torrey, Faith Dickerson

**Affiliations:** 1 Stanley Division of Developmental Neurovirology, Department of Pediatrics. Johns Hopkins School of Medicine, Baltimore Md, United States of America; 2 Stanley Medical Research Institute, Chevy Chase, Md, United States of America; 3 Department of Psychology, Sheppard Pratt Health System, Baltimore Md, United States of America; Hitit University, Faculty of Medicine, TURKEY

## Abstract

A possible role for *Toxoplasma gondii* in the etiopathogenesis of schizophrenia is supported by epidemiological studies and animal models of infection. However, recent studies attempting to link Toxoplasma to schizophrenia have yielded mixed results. We performed a nested case-control study measured serological evidence of exposure to Toxoplasma gondii in a cohort of 2052 individuals. Within this cohort, a total of 1481 individuals had a psychiatric disorder and 571 of were controls without a psychiatric disorder. We found an increased odds of Toxoplasma exposure in individuals with a recent onset of psychosis (OR 2.44, 95% Confidence Interval 1.4–4.4, p < .003). On the other hand, an increased odds of Toxoplasma exposure was not found in individuals with schizophrenia or other psychiatric disorder who did not have a recent onset of psychosis. By identifying the timing of evaluation as a variable, these findings resolve discrepancies in previous studies and suggest a temporal relationship between Toxoplasma exposure and disease onset.

## Author summary

The protozoan parasite *Toxoplasma gondii* has been previously associated with an increased risk of serious psychiatric disorders such as schizophrenia. However, this association has been found in some studies and not others. We examined whether the differences among previous studies might be explained by the timing of patient evaluation and testing. We found that individuals who were evaluated soon after the onset of psychosis had increased odds of exposure to *Toxoplasma gondii* as evidenced by the measurement of antibodies in their blood. However. we did not find an increased rate of exposure to *Toxoplasma gondii* in individuals who had a diagnosis of schizophrenia or bipolar disorder but who did not have recent onset psychosis. Our findings are consistent with Toxoplasma exposure occurring around the time of onset of psychiatric symptoms in individuals with schizophrenia. Our findings might lead to the evaluation of new methods for the early treatment of schizophrenia in some individuals.

## Introduction

*Toxoplasma gondii* is an apicomplexan protozoan with a worldwide distribution. Felines serve as definitive hosts for Toxoplasma and can support the complete life cycle of the organism including sexual reproduction and the shedding of oocysts in the feces. Most other species of warm-blooded animals support the replication of portions of the Toxoplasma life cycle including asexual reproduction and the development of tissue cysts in multiple organs including the brain. Humans can become infected with Toxoplasma through the ingestion of oocysts shed from cats into the environment or by the consumption of tissue cysts in the meat of infected food animals such as pigs, sheep, and cows. Human fetuses can also become infected through vertical transmission from the mothers, although this mode of transmission is relatively rare compared to the other modes of transmission.

As in the case of other intermediate hosts, initial exposure to Toxoplasma in humans can lead to the formation of tissue cysts in multiple organs including the brain. Exposure also leads to a vigorous immune response evident from the presence of specific antibodies directed at Toxoplasma proteins. Previously, tissue cysts were not thought to cause symptoms in immune competent hosts. However recently studies have documented that tissue cysts are engaged in active metabolism and interaction with the host [1]. Accordingly, serological studies indicate that exposure to Toxoplasma can be associated with eye disease [2] as well as an increased risk of a range of neuropsychiatric diseases including schizophrenia[3, 4], bipolar disorder[5], suicidal behavior[6], anxiety disorder [7] and cognitive decline in the elderly [8] in some populations. The factors which determine why some Toxoplasma exposed individuals develop medical or psychiatric disorders while others are unaffected have not been determined but may related to the strain of Toxoplasma, host factors, or the timing of infection.

Schizophrenia is the psychiatric condition that has been most extensively studied in terms of association with *Toxoplasma gondii*. An association between Toxoplasma and schizophrenia was first made in 1953 [9] and has been the subject of numerous studies. A recent meta-analyses consisting of 38 studies reported pooled odds ratios of 2.71 (95% confidence interval 1.93–3.78) relating Toxoplasma exposure to prevalence of schizophrenia. Many of the studies included in the meta-analysis involved individual living in areas with a high prevalence of Toxoplasma exposure (>20% of the adult population) and who were recently hospitalized. [10]

However recently a number of studies have also been reported which did not find a significant association between serological evidence of Toxoplasma and risk of schizophrenia [11–13].A meta-analysis reporting a lower corrected odds ratio has also been recently published [14]. These studies generally involved individuals living in environments with low prevalence of Toxoplasma who had long-standing schizophrenia and were receiving antipsychotic medications. The latter point is of interest since some of the more recently available antipsychotic medications have the ability to inhibit the replication of Toxoplasma organisms.[15, 16]

We postulated that, in areas of low Toxoplasma prevalence, individuals with the recent onset of the symptoms of schizophrenia would be more likely to have evidence of exposure to Toxoplasma than individuals with established schizophrenia who are receiving antipsychotic therapy.

## Methods

### Study participants and procedures

The study population consisted or 2052 individuals with recent onset psychosis, schizophrenia, bipolar disorder, major depressive disorder, or non-psychiatric controls who were enrolled during the period January 1999 through May 2017 in the Stanley Research Program at Sheppard Pratt, Baltimore, Maryland, USA. This cohort, which is ongoing, has been recruited for the study of the association between infection, immunity, and psychiatric disorders. Detailed descriptions of the methods employed for the recruitment and analysis of the cohort have been as previously described [17–19]. The studies were approved by the Institutional Review Boards of the Sheppard Pratt Health System and the Johns Hopkins Medical Institutions following established guidelines. All participants provided written informed consent after the study procedures were explained.

The inclusion criterion for recent onset psychosis was the onset of psychotic symptoms for the first time within the past 24 months defined as the presence of a positive psychotic symptom of at least moderate severity that lasted through the day for several days or occurred several times a week and was not limited to a few brief moments and which was not substance-induced. Participants meeting the criteria for a recent onset of psychosis could have a DSM-IV diagnosis[20] from among the following: schizophrenia; schizoaffective disorder; schizophreniform disorder; psychotic disorder not otherwise specified; brief psychotic disorder; delusional disorder; bipolar I disorder, most recent episode depressed; bipolar I disorder most recent episode manic; bipolar I disorder, most recent episode mixed; single manic episode; bipolar II disorder; major depressive disorder, recurrent; major depressive disorder single episode. Individuals with recent onset psychosis were further divided into those with affective psychosis, defined as having a diagnosis of bipolar disorder or major depressive disorder, and those with non-affective psychosis, defined as having a diagnosis of a schizophrenia-spectrum disorder

The inclusion criterion for individuals with schizophrenia was a diagnosis of schizophrenia, schizophreniform disorder, or schizoaffective disorder. The inclusion criterion for individuals with bipolar disorder included a diagnosis of bipolar I disorder, bipolar II disorder, or bipolar disorder not otherwise specified. Those with major depressive disorder had either a single episode or recurrent episodes. Participants who met the criteria for recent onset of psychosis and another diagnosis were assigned to the recent onset group.

The psychiatric participants were recruited from inpatient and day hospital programs of Sheppard Pratt and from affiliated psychiatric rehabilitation programs. The diagnosis of each psychiatric participant was established by the research team including a board-certified psychiatrist and based on the Structured Clinical Interview for DSM-IV Axis 1 Disorders [20] and available medical records.

The inclusion criterion for the non-psychiatric control individuals was the absence of a current or past psychiatric disorder as determined by screening with the DSM-IV Axis I Disorders, Non-patient Edition [20]The controls were recruited from the same geographic area as the psychiatric participants.

Participants in all groups met the following additional criteria: age 18–65 (except the control participants who were aged 20–60); proficient in English; absence of any history of intravenous substance abuse; absence of intellectual disability by history; absence of HIV infection; absence of serious medical disorder that would affect cognitive functioning; absence of a primary diagnosis of alcohol or substance use disorder per DSM-IV criteria. The occurrence of psychosis not of recent onset was not an exclusion criterion for individuals in the bipolar disorder or major depression diagnostic groups.

All participants were individually administered a brief cognitive battery, the Repeatable Battery for the Assessment of Neuropsychological Status (RBANS) Form A at the study visit. This battery measures a range of domains and yields a scaled Total Score with a nominal population mean of 100.[21], Participants were asked about demographic variables including maternal education as a proxy for pre-morbid socioeconomic status and were asked about their current smoking status. All of the psychiatric participants were also interviewed and rated on the Brief Psychiatric Rating Scale (BPRS).[22] Psychiatric participants were also categorized as to whether or not they had a history of substance abuse (apart from nicotine or caffeine) based on their response to interview questions about the use of alcohol and drugs and on the medical record. Medications received at the time of study enrollment for psychiatric participants were based on the medical chart and self-report. Other demographic and clinical data including illness duration at the time of study entry were obtained by self-report or review of the medical record.

### Laboratory testing

Each participant had a blood sample obtained, generally at study enrollment. For 1875 (91.4%) if the study individuals the sample was obtained within 90 days of intial screening. Plasma was separated from the blood sample by centrifugation and stored at -70 until testing. At the time of testing, the sample was thawed and test for IgG class antibodies to *Toxoplasma gondii* using solid phase enzyme immunoassay as previously described. Assay reagents were obtained from IBL America, Minneapolis Minn. A standard sample with a known amount of antibody was also analyzed in each assay run. For each antibody measurement, a sample was considered to be reactive if it generated a signal which was at least 0.8 times the value generated by the standard as previously described[18], corresponding to approximately 10 International Units (IUs).

### Statistical analyses

Univariate analyses were performed by means of analysis of variance for continuous variables and Pearson’s chi square for categorical variables. The odds ratios associated with seropositivity and clinical diagnosis was calculated by the use of logistic regression models employing age, gender, race, maternal education (as a marker of socioeconomic status), and place of birth (United States or Canada vs other countries). These covariates were selected since they have been previously shown to be associated with the prevalence of antibodies to Toxoplasma and other infectious agents. Missing data were added by imputation. All analyses were performed by STATA Version 12, College Station, Texas.

### Ethics statement

This research was approved by the Institutional Review Boards of the Sheppard Pratt Health System and the Johns Hopkins School of Medicine. All participants were at least 18 years of age and provided written informed consent.

## Results

There were a total number of 2052 individuals in the study population. These included 221 individuals with the recent onset of psychosis, 752 individuals with established schizophrenia, 444 individuals with bipolar disorder without a recent onset of psychosis, 64 individuals with major depressive disorder without a recent onset of psychosis, and 571 control individuals without a psychiatric disorder. Of the 221 individuals with recent onset psychosis, 206 (93.2%) were hospitalized at the time of study recruitment, either in the inpatient service or day hospital. Of the 752 individuals with schizophrenia without a recent onset of psychosis, 141 (18.8%) were hospitalized at the time of recruitment, Of the 444 individuals with bipolar disorder without the recent onset of psychosis, 281 (63.3%) were hospitalized at the time of recruitment. A total of 59 (92.2%) of the 64 individuals with major depressive disorder without the recent onset of psychosis were hospitalized at the time of recruitment. The demographic and clinical characteristics of the population are depicted in Table 1 and Table 2.

**Table 1:** Demographic characteristics of study population.

	Control N = 571	ROP N = 221	Schizophrenia N = 752	Bipolar Disorder N = 444	Major Depression N = 64	Total N = 2052	P*
Age	32.7(11.3)	25.5(7.1)	41.6(11.1)	37.5(14.2)	37.8(14.5)	36.4(12.5)	< .001
Gender-Female	62.7%	42.1%	38.0%	71.6%	62.5%	53.4%	< .001
Race-White	56.6%	56.6%	59.4%	74.6%	70.3%	61,9%	< .001
Maternal Education (Years)	13.6 (2.7)	14.1 (2.8)	12.6 (2.6)	13.3 (2.9)	13.5(3.2)	13.3(2.8)	< .001
Birth-US/Canada	91.1%	90.1%	95.7%	95.7%	95.3%	93.5%	< .003
Pet Cat at Birth	11.9%	15.3%	7.0%	11.3%	12.5%	11.2%	< .038
Pet Cat Before Age 5	16.8%	22.6%	10.9%	19.6%	18.7%	17.3%	< .004
Toxoplasma IgG Positive	6.1%	10.0%	11.2%	11.3%	7.8%	9.6%	< .017

Numbers in parentheses indicated standard deviation.

*P values calculated by analysis of variance for continuous variables and Chi^2^ for discrete variables.

ROP signifies individuals with the recent onset of psychosis.

The groups Schizophrenia, Bipolar Disorder and Major Depression refer to individuals with these diagnosis who did not have a recent onset of psychosis.

**Table 2:** Clinical characteristics of study population.

	ROP N = 221	Schizophrenia N = 752	Bipolar Disorder N = 444	Major Depression N = 64	Total	P*
BPRS Score	46.6(9.3)^1^	45.1(8.8)	44.9(9.4)	48.7(8.3)	45.4(9.1)	< .003
RBANS Total Score	71.8(14.8)	65.4(13.1)	78.2(14.3)	82.5(12.0)	70.9(15.0)	< .001
Age of Onset Years^2^	24.2(7.4)	20.7(7.8)	17.4(9.1)	17.0(9.0)	20.1(8.5)	< .001
Duration^3^ (Years)	0.11 (0.3)	20.6(10.8)	19.8(12.4)	19.8(14.1)	17.5(12.7)	< .001
Atypical Antipsychotic	88.2%	82.6%	64.9%	27.5%	76.2%	< .001
Olanzapine	17.7%	21.5%	12.2%	0	18.7%	< .001
Risperidone	38.9%	25%	16.4%	6.3%	23.7%	< .001
Valproate	13.1%	22.2%	33.7%	0	23.4%	< .001

The groups Schizophrenia, Bipolar Disorder and Major Depression refer to individuals with these diagnosis who did not have a recent onset of psychosis.

^1^.Values represent mean (1SD)

^2^.Onset measures as missing from 4 individuals with established schizophrenia and 1 individual with established bipolar disorder.

^3^.Duration of ROP group in months, 3.6(1.3). Duration data in Bipolar and Major Depression groups are based on the duration of mood symptoms at the time of study entry.

*P values calculated by analysis of variance for continuous variables and Chi^2^ for discrete variables

**Fig 1:**
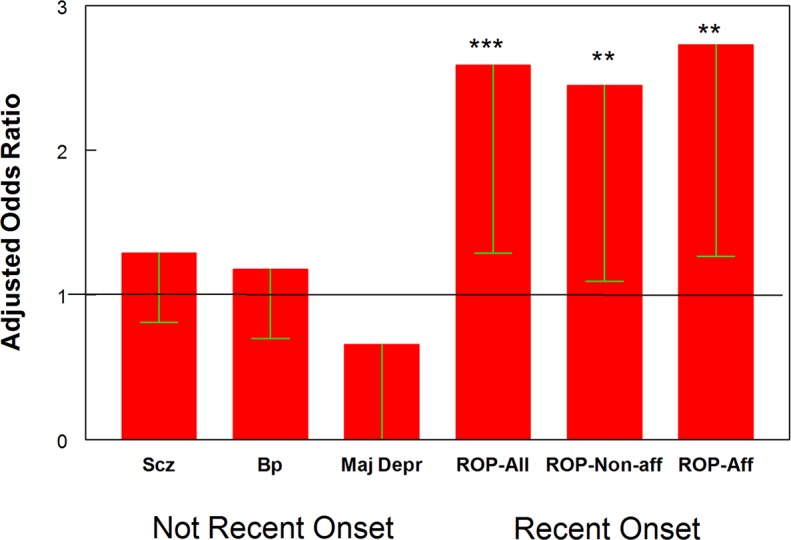
Odds ratios associated with toxoplasma exposure by clinical diagnosis. Bars represent odds ratios (Mean-95% confidence interval) associated with the indicated clinical diagnosis calculated by logistic regression as described in the text with age, gender, race, maternal education, and place of birth as covariates The abbreviations used are as follows: Scz = Non-recent Schizophrenia, Bp = Non-recent Bipolar Disorder; Maj Depr = Major Depressive Disorder without Recent Onset Psychosis; ROP-All = Recent Onset Psychosis, All Cases; ROP-Non-aff = Recent Onset Psychosis, Individuals with Non-Affective Psychosis; ROP-Aff = Recent Onset Psychosis, Individuals with Affective Psychosis,. *** p < .003**p < .03. Not recent onset refers to individuals who did not have recent onset of psychosis.

As noted in Table 1, the overall prevalence of Toxoplasma IgG antibodies in the study population was 9.6%. Unadjusted analysis of variance indicated a significant difference among the diagnostic groups (chi^2 =^ 11.8, p < .017). This difference was further explored by means of logistic regression models employing age, gender, race, maternal education (as a measure of socioeconomic status) and birth outside of the United States or Canada. As depicted in Fig 1, this analysis indicated that Toxoplasma exposure was associated with recent onset psychosis with an Odds Ratio of 2.44 (95% Confidence Interval 1.36–4.38, p < .003). The odds of association with Toxoplasma exposure were similar in the subgroups of individuals with recent onset psychosis who had primarily non-affective (2.49, 95% CI 1.19–5.22, p < .015) or affective psychosis (2.40, 95% CI 1.15–4.00, p < .019). Toxoplasma exposure was associated with somewhat increased odds of being in the established schizophrenia or bipolar groups but these odds did not differ significantly from the controls. In this model, the prevalence of Toxoplasma was also associated with increasing age (p < .001) and birth outside of the United States or Canada (p < .007) but not with gender, race, or maternal education. Within the group of individuals with recent onset psychosis Toxoplasma exposure was associated with receiving the drug olanzapine (chi2 = 6.7, p < .01) but was not associated with receipt of other medications, BPRS symptom score, RBANS cognitive score, cigarette smoking or a history of drug or alcohol abuse.

## Discussion

We found that individuals with recent onset psychosis had a significantly increased rate of exposure to *Toxoplasma gondii* as determined by antibody measurement. This increase was independent of demographic factors associated with Toxoplasma exposure such as age, place of birth and socioeconomic status. The odds ratio associated with Toxoplasma exposure in our population with recent onset psychosis was, 2.44 (95% Confidence Interval 1.36–4.38). This odds ratio is similar to that reported in some recent meta-analyses examining the association between Toxoplasma and recent onset psychosis and schizophrenia [10, 23]. It is of note that some of these meta-analyses included studies of individuals with both recent onset psychosis and established schizophrenia. However, in many cases the prior treatment status of the study individuals was not reported.

On the other hand, the level of Toxoplasma exposure in our population of individuals with established schizophrenia or bipolar disorder, while somewhat elevated, did not differ significantly from that of the control population. It is of note that the individuals with these disorders were derived from the same geographic area and tested by the same methods as those employed for the analysis of individuals with recent onset psychosis, suggesting that these differences are not related to socio-demographic factors.

The reasons for finding an increased rate of Toxoplasma exposure in individuals with recent onset psychosis but not established schizophrenia is not known with certainty but may be related to changes in antibody levels over time. It had previously been thought that Toxoplasma seropositivity was lifelong. However this concept has been called into question on the basis of longitudinal and population based analyses [24, 25] leading to the suggestion that persistent exposure to Toxoplasma is required for the maintenance of antibody levels. It is thus possible that antibody levels in individuals with recent onset psychosis fall over time in the absence of re-exposure to the point that they are not different from those of controls when assessed many years following disease onset. Valproic acid and other medications used for the treatment of schizophrenia or bipolar disorder have been shown to have anti-Toxoplasma activity in cell culture[16]. This activity may contribute to the decline in Toxoplasma seropositivity over time. It is of note in this regard that this process may take several years since a previous study indicated that samples obtained within 1 year of diagnosis did not show significant changes in Toxoplasma antibody levels.[26] It is also of note that we found an association between Toxoplasma seropositivity within the group of individuals with recent onset psychosis and treatment with the second generation anti-psychotic medication Olanzapine. The reasons for this association are not known with certainty but may be related to a differential effect of this medication on Toxoplasma infection as compared to other anti-psychotic medications within the central nervous system [15, 16].

Our finding that individuals with established schizophrenia or bipolar disorder did not have increased odds of having Toxoplasma exposure is consistent with several other studies performed in low prevalence populations where individuals were receiving medications for extended periods of time. Our findings thus serve to resolve some of the discrepancies in past studies based on differences in regard to the timing of illness onset and assessment for Toxoplasma exposure as well as the receipt of medications. Prospective longitudinal studies relating to the timing of exposure to Toxoplasma and the onset of psychiatric disorders are necessary to directly address the issue of the temporal relationship between Toxoplasma exposure and subsequent risk of psychiatric disorders. These studies should include measurements of additional class-specific and subclass specific measures of antibodies, such as measurements of IgM and IgA class antibodies and measurements of IgG subclasses, which were not available from all members of this study population.

It is of note that the prevalence of Toxoplasma is decreasing in many areas of the world. In the case of our study population, the prevalence of exposure to Toxoplasma in the control population was 6.1%, a level consistent with recent studies of young adults living in the United States.[27] The reason for the decrease in prevalence of Toxoplasma exposure is not known with certainty but may be related to improved levels of food preparation and water purification.

The effect of this decrease in Toxoplasma exposure on human diseases relating to Toxoplasma is unclear. On the one hand, the rate of these diseases may decrease due to a lower level of organisms in the environment. On the other hand, a lower level of exposure in childhood may result in a larger number of individuals who are susceptible to primary infection in later life, resulting in an increased incidence of adult onset disorders. The long-term effects of these epidemiological changes on the role of Toxoplasma on human health and human disease are thus worthy of close examination.

Unlike Toxoplasma infection in immune deficient individuals which is associated with the rapid replication of the tachyzoite form of the organism, Toxoplasma infection in immune competent hosts is associated with slowly replicating tissue cysts which are relatively resistant to currently available anti-Toxoplasma medications [28]. Recently methods have been developed for the treatment of Toxoplasma tissue cysts within the brains of experimentally infected animals[29]. In addition, novel immunogenic modalities have been developed for the prevention of experimental Toxoplasma infection [30]. The finding of a definitive association between Toxoplasma exposure and human psychiatric disorder in immune competent individuals might provide the framework for the further development of these interventions and their application to the prevention and treatment of Toxoplasma associated brain disorders.
